# Molecular MRI of acute necrosis with a novel dna-binding gadolinium chelate: kinetics of cell death and clearance in infarcted myocardium

**DOI:** 10.1186/1532-429X-13-S1-O23

**Published:** 2011-02-02

**Authors:** Shuning Huang, Howard H Chen, Hushan Yuan, Guangping Dai, Daniel Schule, Soeun Ngoy, Ronglih Liao, Peter Caravan, Lee Josephson, David Sosnovik

**Affiliations:** 1 Martinos Center for Biomedical Imaging, Massachusetts General Hospital, Charlestown, MA, USA; 2 Center for Translational Nuclear Medicine and Molecular Imaging, Massachusetts General Hospital, Charlestown, MA, USA; 3 Cardiology Division, Brigham and Woman’s Hospital, Boston, MA, USA

## Objective

In the current study, we describe a novel approach to image acute necrotic cell death in vivo through the use of a DNA-binding gadolinium chelate (Gd-TO).

## Background

Detecting cell death in vivo is extremely important in understanding disease pathogenesis, assessing disease progression, and evaluating treatment efficacy. Various MRI techniques have been developed over the years to image cell death. However, most of these techniques are based largely on non-specific pharmacokinetics and accumulation, and usually cannot discriminate acute and chronic injury. Here we present a novel DNA-binding gadolinium chelate (Gd-TO) that binds specifically to the exposed DNA of ruptured/necrotic cells. We show that Gd-TO can be used to selectively detect and image the kinetics of acute necrotic cell death and the subsequent clearance of necrotic debris from injured tissue.

## Material and methods

In vivo imaging was performed in twenty infarcted (permanent ligation of the left coronary artery) C57BL6 mice. The infarcted mice were injected with 0.1 mmol/kg of Gd-TO (15 mice) or Gd-DTPA (5 mice) at varying times post-infarction. MRI was performed 2-3 hours after probe injection on a 9.4T scanner (Biospin, Bruker) with a gradient strength of 150 Gauss/cm. A modified cardiac gated Look-Locker FISP sequence (TR: 3000ms, TE 1.3, MTX: 160x160, FOV: 2.5 x 2.5 cm^2^) was used to detect changes in signal intensity and the longitudinal relaxation rate (R_1_) in the infarcted myocardium. R_1_ maps of the myocardium were constructed using Matlab (Mathwork). Statistical analysis (unpaired t-test, ANOVA, and a Tukey’s post-test comparison) of the data was performed with Prism (Graphpad).

## Results

**Figure 1:**
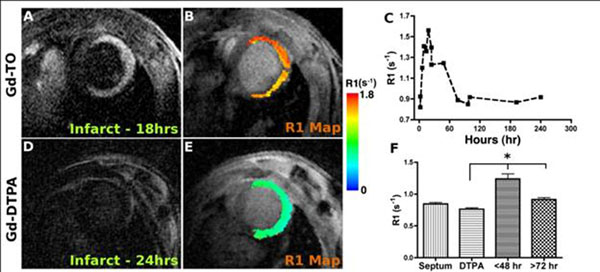
MRI of mice with acute (,24 hours) myocardial infarction injected with either (A,B) Gd-TO or (D, E) Gd-DTPA. MRI was performed 2-3 hours after injection. High levels of Gf-TO accumulate in acute infarcts producing signal hyperintensity on invertsion recovery images (A) and high R1 values (B). In contrast, within 2-3 hours of injection Gd-DTPA has completely washed out of the infarct and produces no change in signal intensity or R1 (D, E). The accumulation of Gd-TO peaks 9-18 hours after infacrtion, and within 72-96 hours has returned to baseline (C). Significant different (*p<0.01) in R1 in the infarct were seen between those mice imaged with Gd-TO within 48 hours of infarction versus more than 72 hours after infarction (F). Likewise, significant differences were seen between mice injected with Gd-TO versus GF-DTPA within 48 hours of infarction (F).

Cell rupture (Gd-TO uptake) was present within 2 hours of infarction, but peaked 9-18hrs after the onset of injury (Fig 1C). No uptake of Gd-TO was seen 72-96 hours after injury, indicating complete clearance of the necrotic debris at this time. Significant differences were seen between mice injected with Gd-TO less than 48 hours post-infarction and those injected more than 72 hours after infarction. Likewise, while Gd-TO was robustly retained in acute infarcts, complete myocardial washout of Gd-DTPA was seen within 60-90 minutes (Fig 1). Fluorescence microscopy showed that Gd-TO was exclusively bound to DNA in the infarct.

## Conclusion

The uptake of Gd-TO delineates a narrow time window during which acutely necrotic cells are present within an injured tissue. This could have important implications for guiding tissue salvage and treatment.

